# An agent-based exploration of the influence of needs on health protection motivation and intentions

**DOI:** 10.3389/fpsyg.2026.1581156

**Published:** 2026-03-24

**Authors:** Patrick Mertes, Veronika Kurchyna, Ye Eun Bae, Jan Ole Berndt, Ingo Timm

**Affiliations:** 1 Cognitive Social Simulation, German Research Center for Artificial Intelligence, Trier, Germany; 2 Business Informatics I, Trier University, Trier, Germany

**Keywords:** agent-based simulation, cognitive dissonance, health behavior, protection motivation theory, social simulation, social smoking

## Abstract

Human behavior plays a crucial role in public health. In the same sense, understanding the multifaceted factors influencing health-related decisions is essential to derive meaningful protective measures. In this study, we demonstrate how values, social norms and individual needs can be combined within the theoretical framework of the Protection Motivation Theory (PMT) to study the intention-behavior gap regarding smoking in social settings. By extending upon a previous model that relied on social pressure as primary mechanism, we investigate how the inclusion of needs shifts behavior toward higher polarization and stronger adherence with intentions. As a novelty compared to previous approaches, conflicts of social pressure and different needs are the source of cognitive dissonance, leading to a gradual shift in attitudes or social network constellations. Most particularly, the inclusion of needs leads to the observation of both peer pressure-induced smoking as well as frequent failure to cease smoking as outcomes in the model.

## 1 Introduction

Human behavior plays a crucial role in public health crises, such as COVID-19, or health-related issues like smoking. To understand behavior in these contexts, it is essential to analyze decisions that go beyond observable actions (Dignum, 2021), considering motivational and emotional states, values, social norms, environmental influences, and individual needs (Dörner et al., 2006; Heidari et al., 2020).

Among those factors, needs are known to drive human behavior, arising from dissatisfaction in different aspects of everyday life and leading to fulfillment-seeking actions (Galtung, 1980). Needs are classified into physiological (survival-related) and psychological (including love, esteem, and self-actualization) needs (Maslow, 1943). While physiological needs are universal, variations in how individuals prioritize psychological needs lead to differences in decision-making (Ghorbani et al., 2021), emphasizing the necessity for taking individual aspects into account.

As an investigation method, agent-based models (ABMs) can effectively simplify and realistically represent such complex systems, particularly in public health (Badham et al., 2018) by providing accurate, computationally efficient simulations (Venkatesan et al., 2019) to gain insights into behaviors that reduce disease risks, such as smoking and alcohol consumption (Tracy et al., 2018). Moreover, they provide a controlled environment for experimenting with psychological theories (Smith and Conrey, 2007). As a subfield in agent-based modeling, social simulation explores individual responses to social influences, personal needs, and innate dispositions. Some models incorporate needs into decision-making processes (Squazzoni et al., 2014; Ghorbani et al., 2021). However, current health risk models often neglect individual needs when applying psychological theories to explain behavior.

Smoking, as social activity, exemplifies social pressure clashing with self-protection motivation (Xu et al., 2015). Despite health risks, social pressure has a significant impact on behavior (Tesser, 1980). To examine the interplay of these conflicting forces, Protection Motivation Theory (PMT) provides a theoretical framework: Protection motivation involves assessing threats and coping ability, making decisions accordingly with broad applicability to health-related behaviors like smoking (Floyd et al., 2000; Kurchyna et al., 2024). Besides social pressure, individual needs further drive actions and decisions (Dignum, 2021)—dissatisfaction of needs prompts individuals to seek fulfillment of them (Taormina and Gao, 2013; Ghorbani et al., 2021). Weighing the influence of social pressure against needs can create a discrepancy between original intention and the actually conducted action caused by conflicting interests. In real life, this so-called intention-behavior-gap between intention and actual behavior is prominent across various domains, including smoking, and unevenly distributed across the types of non-adherence to original intentions (Orbell and Sheeran, 1998). As such, it's not only important to consider the width of the gap — but also the distinction of who is actually deviating.

The aim of this work is to examine the intention-behavior gap by observing how individual behaviors are influenced within social networks using the PMT as a decision-making framework and integrating needs building upon an existing ABM (Kurchyna et al., 2024). We extend the model to include fluctuating needs through a water-tank model (Dörner et al., 2006), which tracks varying levels of needs at a given point of time. We believe that encorporation of individual needs will enable a more flexible decision-making of agents as individuals may have varying prioritization of needs and thus, such a model better reproduces real-life phenomenons compared to the original model without dynamic needs influencing decision-making.

## 2 Theoretical foundations and related works

### 2.1 Needs and values

Needs are fundamental, universal requirements that individuals seek to fulfill for survival, wellbeing, and growth (Taormina and Gao, 2013) and they serve as key motivators for actions (Galtung, 1980). While different theories exist to offer insight into the human mind (Osemeke and Adegboyega, 2017), Maslow's hierarchy of needs is chosen for this study due to its broad recognition and significance in research (Wahba and Bridwell, 1976). In this theoretical framework, needs are organized hierarchically, like a pyramid, wherein lower-level needs must be satisfied before higher-level needs can be addressed. These needs are typically structured as follows (Maslow, 1943):

1. **Physiological needs**: The foundation of Maslow's hierarchy, these include essential survival requirements like air, food, water, shelter, sleep, and clothing. Addressing these needs is crucial for overall wellbeing (McLeod, 2007).
2. **Safety**: After physiological needs are met, safety needs take precedence, focusing on protection from threats like harm, disease, and instability. Satisfying these needs involves ensuring physical and emotional security.
3. **Love and belonging**: Once safety needs are fulfilled, the need for interpersonal relationships and social belonging arises. This includes family, friendship, intimacy, and acceptance within social groups.
4. **Esteem**: These involve the need for self-esteem and the esteem of others, encompassing desires for strength, achievement, and respect. Meeting these needs fosters self-confidence and a sense of usefulness.
5. **Self-actualization**: The highest level, these needs relate to realizing one's potential and pursuing personal growth. Self-actualization is the process of becoming one's true self, with individual expressions varying widely.

In reality, the hierarchy of needs proposed by Maslow is not as rigid as described above. Their order is flexible depending on prevailing external circumstances or individual differences (Maslow, 1943). To account for such variance, values serve as key motivators: they are key principles that shape attitudes, behavior, and decision-making processes. Although the proposed values may be universal, individuals tend to significantly differ when assigning relative importance and priorities, which serves as starting point for the comparison of behavior (Heidari et al., 2020). They characterize cultural groups, societies, and individuals, enabling the tracking of changes over time and explaining motivations behind specific attitudes and behaviors (Schwartz et al., 2012).

These basic human values, as proposed by Schwartz et al. (2012), are generally grouped into four categories, even though there is a continuum of related motivations that blurs clear distinctions:

1. **Self-transcendence** includes universalism and benevolence as values, and emphasizes support of others beyond one's own interests.
2. **Conservation** summarizes security, conformity and tradition as the maintenance of the status-quo and the resistance to change and uncertainty associated with it.
3. In contrast, **Openess to change**, consisting of self-direction, stimulation and hedonism, is the pursuit of novel experiences, independence and change.
4. **Self-enhancement**, as opposed to self-transcendence, emphasizes achievement and power (as well as hedonism) and the selfish goals and benefits associated with the pursuit of these values.

Values serve as a critical mechanism for prioritizing competing needs among diverse agents (Ghorbani et al., 2021); consequently, they are utilized in this study to govern the prioritization of agent requirements. Although personal values undergo gradual shifts over the life course (Van de Poel, 2021), longitudinal research indicates that rank-order stability remains remarkably high (Vecchione et al., 2016). This suggests that while absolute value scores may fluctuate, individuals typically maintain a consistent relative standing compared to their peers and rarely exhibit radical shifts between opposing value poles. Furthermore, empirical evidence demonstrates that value stability is even more pronounced over shorter durations, such as three-month intervals (Bardi et al., 2009). Given that the current simulation encompasses 150 interactions—representing a temporal window of no more than a few months—the underlying values are modeled as constant parameters, as further detailed in Section 3.4.1.

### 2.2 Watertank model of needs satisfaction

As described previously, values offer a conceptual basis for the different prioritization of needs. In terms of implementation, the watertank model by Dörner et al. (2006) provides an intuitively simple representation of needs: tanks with varying water levels represent degrees of satisfaction or dissatisfaction (Ghorbani et al., 2021). Each tank has a fixed capacity, a satisfaction level, and a threshold that signals when a need becomes noticeable, and should be prioritized. Depletion occurs over time, representing a gradual decline in the satisfaction level (Heidari et al., 2020).

The depleted needs are satisfied by engaging in relevant activities that replenish the tanks. Individuals choose actions based on anticipated satisfaction, prioritizing the most urgent needs, often without considering long-term effects such as health, relationships or future needs (Ghorbani et al., 2021). When a need's satisfaction level falls below its threshold, motivation is triggered. Multiple needs often compete, and the need with the highest anticipated satisfaction value guides behavior (Dörner et al., 2006). This prioritization can align with Maslow's hierarchy, focusing on needs at the base of the pyramid first, or vary based on threshold deviation and personal values (McLeod, 2007). Needs with a high threshold are seen as more important, making individuals more sensitive to fluctuations in their satisfaction (Ghorbani et al., 2021). The need driving behavior is constantly reassessed, allowing shifts in motivation (Dörner et al., 2006).

### 2.3 Protection motivation

Confronting disease risks often prompts individuals to adopt protective behaviors to mitigate these risks (Abdulkareem et al., 2018). PMT is a psychological model that explains behavioral changes as a function of both internal and external influences. It is widely applied to various health and protection-related behaviors (Floyd et al., 2000). It focuses on two cognitive processes: threat appraisal and coping appraisal, which guide whether an individual adopts adaptive (healthy) or maladaptive (unhealthy) behavior (Rogers, 1983). In order to assess the two appraisals, it identifies intrinsic factors—such as personal attitudes and self-efficacy (the belief in one's ability to perform protective actions like quitting smoking)—and extrinsic factors, including situational conditions and societal influences like peer pressure and social norms (Rogers, 1983).

More specifically, threat appraisal involves assessing the risk of harmful behavior, influenced by perceived severity and vulnerability. High intrinsic rewards (e.g., pleasure) or extrinsic rewards (e.g., social acceptance) can increase the likelihood of maladaptive responses, like smoking. Furthermore, coping appraisal evaluates the effectiveness of protective actions and self-efficacy. Higher efficacy promotes healthier behaviors, while response costs (e.g., effort or disruption) can hinder them (Rogers, 1983).

The theory operates as an additive model, where factors increasing or decreasing the likelihood of adaptive or maladaptive behavior combine to determine the final outcome. These adaptive or maladaptive responses can consist of direct reactions, repeated actions, or inhibition of harmful behavior (Rogers, 1983).

### 2.4 Social pressure and cognitive dissonance

As seen above, decision-making is influenced by both intrinsic and extrinsic factors. In the context of smoking, such extrinsic influences stem from the social environment (Xu et al., 2015). For example, social pressure plays a significant role, manifesting as expectations from one's immediate social circle, which can compel individuals to conform or resist, potentially facing consequences for nonconformity (Tesser, 1980), such as social exclusion. This pressure can lead to cognitive dissonance, a state of internal discomfort when personal beliefs conflict with social expectations (Festinger, 1957).

To reduce cognitive dissonance, individuals may adopt socially desirable behaviors, such as quitting smoking when surrounded by non-smokers. Alternatively, they might maintain their behavior despite social pressure, or seek out like-minded individuals to reduce their cognivie dissonance (Festinger, 1954). Over time, repeated exposure to social pressure can gradually align an individual's disposition with the prevailing social norms, influencing future behavior in similar situations.

### 2.5 Agent-based modeling and related work

Agent-based modeling helps to understand complex systems by simulating individuals as autonomous agents with behaviors and interactions based on set rules. These agents adapt and make decisions as their environment changes, leading to emergent effects (Macal and North, 2005), such as polarization or segregation as common observations. The model simplifies reality by focusing on essential aspects, making it useful for exploring behaviors and interactions, especially in public health contexts (Badham et al., 2018).

This study builds on previous research by Kurchyna et al. (2024), which extensively explored the integration of PMT and social pressure within an ABM. In the study, agents experience social pressure from their immediate social environment and make decisions about health-related actions (e.g., whether to smoke) based on the assessment of protection motivation in the context of PMT. PMT serves as the processing mechanism for various influences—balancing the agent's personal attitudes and health on one hand, and social influences from peers and the local environment on the other. Based on these factors, agents decide between adaptive and maladaptive behaviors to resolve conflicts of their own attitude and the perceived norms in their surroundings.

The model in the previous work captures static needs based on actor archetypes, without yet modeling the gradual build-up of dissatisfaction or its mitigation through resolution of cognitive dissonance. In the experiments, no particular attention was paid to intentions—while most agents remained part of their initial group (smoker or non-smoker), there was no examination of how many of the agents would have chosen otherwise in a dynamic setting where needs are dynamic, not a mere source of initial preferences.

Prior to the implementation of the extended model, commonly used methods of modeling an agent's internal state and needs were examined:

1. In the **goal-oriented** approach, agents pursue specific goals that shape their behavior, but this can lead to rigid and unrealistic actions, especially when managing multiple goals (Dignum, 2021).
2. The **utility-based** approach uses utility functions to measure preferences and optimize decisions. However, it assumes static preferences, which can result in unrealistic behaviors when circumstances change (Russell and Norvig, 2010), such as scarcity, diminishing returns or novel alternatives.
3. Given the limitations of these two methods, the **water tank model** offers an alternative by controlling behavior over time and adapting to shifting priorities based on the context (Ghorbani et al., 2021).

Such approaches have been used successfully to investigate social and spatial impacts of behaviors on public health. Besides the previous work by Kurchyna et al. (2024), there exist other agent-based examinations of smoking with a focus on needs, but without explicit psychological theories explaining health behavior (Di Tosto and Dignum, 2012) or with a focus on acute crises, such as pandemics (Dignum et al., 2021). Since there is empirical evidence that PMT is an appropriate theoretical framework to explain behavior in the context of smoking (Lin et al., 2022), it remains the guiding framework.

## 3 Concept and model description

During the simulation, the agents move randomly in a continuous space in each step. Each agent has a set number of close friends [default parameter value: 2 (McPherson et al., 2006)], with persistent ties regardless of movement - these friends are the social network, initialized randomly at the beginning of the simulation. The local network consists of all nearby agents (default distance: 6 patches) and it changes after each step. This design mimics everyday situations where people meet others throughout their day-to-day activities, yet physically absent peers still exert an influence on their behavior (Allen and Wilder, 1979).

**Figure 1:**
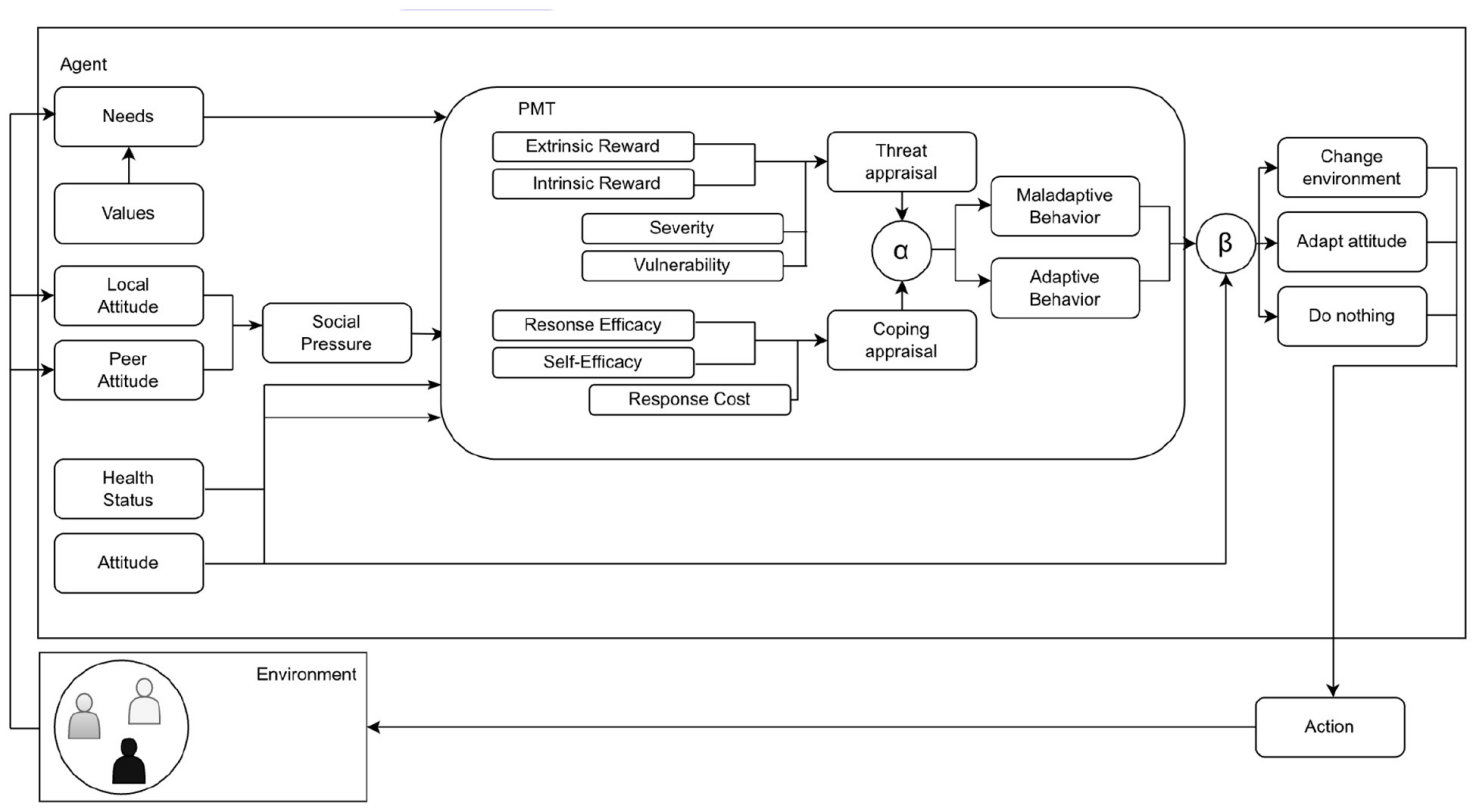
Model of needs, protective effort and social pressure according to Kurchyna et al. (2024).

This study expands the original model to include individual needs in decision-making on adaptive or maladaptive behavior. As shown in Figure 1, the model focuses on the internal mechanisms of agents, rather than the external events agents react to. While the cognitive processes are modeled in detail, the environment and actions within it is a minimalistic abstraction. Social pressure is a major contributor to changes in the Protection Motivation of agents, as examined by Kurchyna et al. (2024) in greater detail.

For the smoking use case, three relevant needs from Maslow's hierarchy, safety, belonging, and self-actualization, are selected to manage model complexity. They are the needs that influence smoking behavior due to factors like health risks, social acceptance, and personal growth, while other needs (e.g., physiological) are excluded to reduce complexity unrelated to the use-case.

This model allows multiple needs to be satisfied simultaneously, although only one need is salient to guide decisions. Prioritization follows Maslow's hierarchy with more basic needs taking precedence.

### 3.1 Foundations for extended components

This section explains the collection and selection of the required data used in the modeling process, forming the foundation for the simulation. Relevant data and previously made assumptions for the original model are still mostly used in this context. Therefore, supporting evidence is needed to implement the water tank approach, represent individual needs and their satisfaction, as well as to extend the PMT.

The concept of fluid level of needs or thresholds determining when a need becomes salient, originate from the works of Di Tosto and Dignum (2012). Important facets concerning need satisfaction were adopted from the approaches of Heidari et al. (2020). Similar to their work, need satisfaction is time-dependent, leading to the decrease in unsatisfied needs' fluid levels by a specific factor after a defined period. Similarly, when the agent takes an action aligned with a specific need, it leads to an elevation in the fluid level. Aligned with the approach presented by Ghorbani et al. (2021), need satisfaction is achieved through engagement in specific activities, wherein the individual selects actions leading to the highest gain in satisfaction of the currently most salient need.

### 3.2 Values and needs for agent archetypes

To determine the prioritization of different needs, agent archetypes based on dominant values are selected, similar to existing approaches (y López et al., 2002; Kurchyna et al., 2024), which define the agent type by a prioritized core value: hedonism, security, or conformity, as defined by Schwartz et al. (2012). Respectively, the prioritized needs align with these values. The need for *Belonging* reflects conformity as prioritized value. *Self-Actualization* is a need that is aligned with hedonism in this simplified model. Finally, a need for *Safety* represents security as major value.

Each agent possesses unique individual values, with the dominant value being determined by the agent's archetype, and these values remain stable over the course of the simulation, as opposed to the original model, in which agents were swayed from their fundamental core beliefs more easily.

The definition of archetypes and the subsequent priorization of different needs is necessary for the modeling of the water tank approach as presented by Dörner et al. (2006), wherein each need is a water tank with fluid levels indicating satisfaction. Each tank has a fixed capacity (set to 1). Notably, almost all introduced variables are defined within the range of [0, 1]. Fluid levels are initialized randomly, with need thresholds derived from agent values (Dignum, 2021). For the randomization, continuous uniform distribution is used. Each need is assigned a threshold value that determines its salience and aids in prioritizing among competing needs. For example, if security-related values are of high importance, the safety need is prioritized, resulting in a higher threshold value. A higher threshold corresponds to a shorter time period for the need to be satisfied.

### 3.3 Individualized watertank model of needs

Over the course of the simulation, these *Fluid Levels* decline based on the model proposed by Heidari et al. (2020). To prevent rapid drops of needs, there is a delay between the satisfaction of a need and the resumption of depletion.

Individuals aim to maintain fluid levels in their tanks that align with designated thresholds or higher. Each tank indicates a need, activated based on the extent of this deviation, prompting the agent to satisfy the need. When a tank's fluid level drops below its threshold, it triggers a behavior that will satisfy the need. Agents often experience necessity of fulfilling multiple needs simultaneously, leading to potential conflicts over which need to prioritize. As literature suggests, behavior is guided by only one salient need at a time (Dörner et al., 2006), and there are two approaches to the selection of priority needs:

1. Based on threshold deviation and a need-specific factor representing the prioritization of needs.
2. Hierarchical approach, where needs are prioritized by their position in the hierarchy of needs.

This model adopts the second approach, prioritizing needs lower in the hierarchy (e.g., safety over self-actualization if both are unsatisfied). When two or more needs are on the same level of hierarchy, needs with higher threshold values are considered more significant, and individuals are more sensitive to changes in their satisfaction levels of those needs. The need driving behavior is monitored regularly, allowing other needs to be prioritized when necessary.

**Figure 2:**
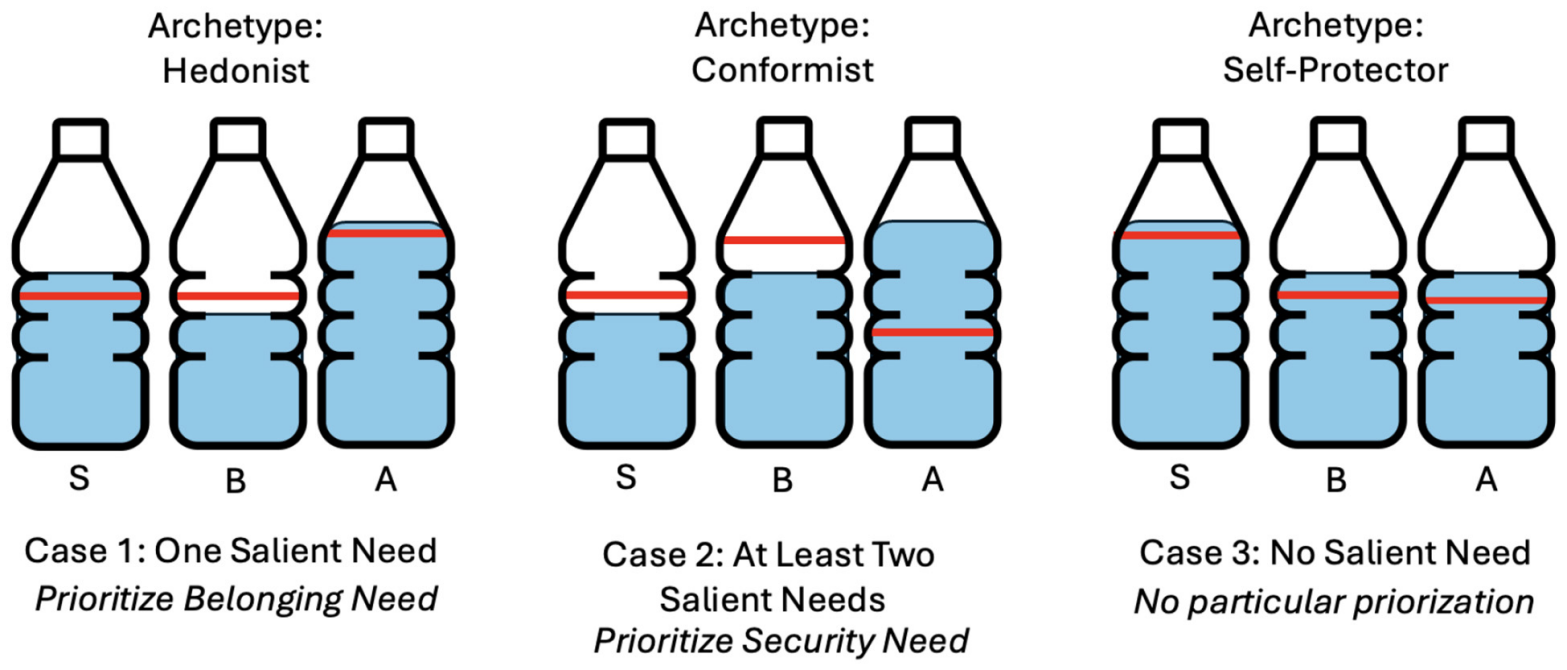
Exemplary illustration of the needs-thresholds for the three archetypes as well as the different cases where salience leads to priorisation of needs. Where 'S' stands for *Safety*, 'B' for *Belonging*, and 'A' for *(Self)-Actualization*.

As shown in Figure 2, the thresholds for salience vary depending on the agent's archetype. As the fluid levels deplete over time and are replenished through appropriate actions, even low-priority needs may become salient and prompt behavior aimed at satisfying the unmet need.

### 3.4 Selection of actions using protection motivation theory

As presented by Kurchyna et al. (2024), agents choose between adaptive and maladaptive behaviors, as defined by PMT. Needs influence these decisions through their impact on the individual components of the PMT based on the prevailing threshold deviation and a logistic function designed to emphasize lower satisfaction levels. This maximization of satisfaction is combined with the hierarchical approach - if two needs are salient, the rank in the hierarchy of needs determines which one takes priority and guides action.

**Figure 3:**
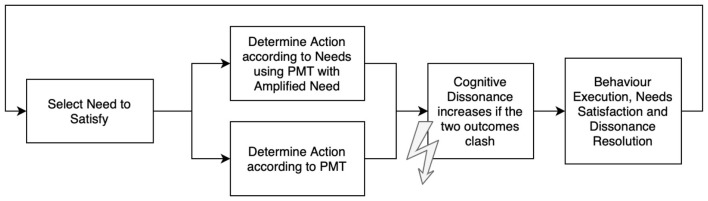
The two-tiered approach contrasts the selection of actions according to PMT and the alternative choice if needs are taken into account. Conflicts in these two evaluations result in growing cognitive dissonance.

In the implemented model, agents identify their salient needs, form intentions to satisfy their most urgent needs, and select an appropriate action to maximize the expected satisfaction. As PMT deals with conflicting behaviors, adaptive, and maladaptive, these intensions may not be translated into actions, leading to the Intention-Behavior-Gap often observed in empirical findings (MOAN and Rise, 2005). Through such conflicts, cognitive dissonance can grow, as shown in Figure 3. Through the two-step approach, instances of an intention-behavior gap can be tracked and analyzed during results analysis.

#### 3.4.1 PMT components

As described in Section 2.3, PMT suggests that individuals choose to conduct a behavior based on two assessments: threat appraisal and coping appraisal. These assessments determine whether an agent's behavior should be adaptive (protective) or maladaptive (harmful) based on those appraisals. The basic components of calculating threat appraisal and coping appraisal have been expanded to incorporate adjustments for three individual needs, belonging, self-actualization and safety. These needs are integrated into the model and influence how threat and coping appraisals are calculated, reflecting how unmet needs affect behavior decisions.

In the **Threat appraisal calculation** (*A*_*t*_), the likelihood of engaging in maladaptive behavior is influenced by several factors, including intrinsic and extrinsic rewards, vulnerability, and severity.

*Intrinsic rewards* (*r*_*i*_) reflect the pleasure or satisfaction an individual derives from smoking, calculated as shown in Equation 1. These rewards are tied to the agent's attitude toward smoking (*a*_*o*_) and are influenced by the need for self-actualization (*n*_*a*_) due to its inclusion of hedonistic aspects associated with smoking. When the self-actualization need is unmet (below the threshold), the intrinsic reward for smoking increases, as smoking is perceived as fulfilling this unmet need for hedonism and pleasure.


$$\begin{array}{l} r_{i}=\frac{a_{o}+n_{a}}{2} \end{array}$$ (1)

*Extrinsic rewards* (*r*_*e*_) are linked to the social pressure in the environment, shown in Equation 2. They depend on the attitudes of the individual's social environment toward smoking (*a*_*s*_). If an agent resides in a smoker-friendly environment or is surrounded by smoking peers, the social reward for smoking increases, due to its need for belonging (*n*_*b*_), making it more susceptible to social pressure. This inclusion of social factors aligns with the idea that smoking can serve to meet social needs such as acceptance and group inclusion (Eiser, 1985).


$$\begin{array}{l} r_{e}=\frac{a_{s}+n_{b}}{2} \end{array}$$ (2)

The *Severity* (*s*_*p*_) reflects the perceived seriousness of the consequences of smoking, grounded in an objective measure (*s*_*o*_) like the case fatality rate from smoking-related diseases (e.g., 64% in Germany). However, individual perceptions of severity vary based on needs, particularly the need for safety (*n*_*s*_) and the need for belonging (*n*_*b*_). A higher need for safety can increase an individual's perception of the severity of smoking-related risks, while need for belonging may prompt a decrease, as shown in Equation 3.


$$\begin{array}{l} s_{p}=s_{o}+\left(\left(1-n_{b}\right)*\left(n_{s}-s_{o}\right)\right) \end{array}$$ (3)

Likewise, *Vulnerability* (*v*_*p*_) indicates how personally relevant the threat of smoking is to the individual. It is influenced by the agent's health status (*v*_*o*_) and their need for safety (*n*_*s*_). If the individual has poor health, or the need for safety is unmet, their perceived vulnerability to smoking-related harm increases, as shown in Equation 4.


$$\begin{array}{l} v_{p}=\frac{n_{s}+\left(1-v_{o}\right)}{2} \end{array}$$ (4)

Overall, the threat appraisal *A*_*t*_ is thus the combination of different factors as shown in Equation 5:


$$\begin{array}{l} A_{t}=\frac{r_{i}+r_{e}}{2}-\frac{s_{p}+v_{p}}{2} \end{array}$$ (5)

During the **Coping appraisal calculation** (*A*_*c*_), the agent assesses their ability to resist engaging in maladaptive behavior (e.g., smoking) and their capacity to adopt protective, adaptive behavior. It focuses on three primary components: self-efficacy, response efficacy, and response costs.

*Self-efficacy* (*e*_*S*_) is the agent's belief in their ability to successfully refrain from smoking. It is influenced by both intrinsic rewards (*r*_*i*_) (personal satisfaction derived from smoking) and social pressure (*a*_*s*_) (nearby agents' attitudes toward smoking). The self-efficacy peaks when these influences are low, meaning the agent feels more confident in quitting smoking when they do not experience strong intrinsic rewards from smoking or when they are not in a smoker-friendly environment.


$$\begin{array}{l} e_{S}=\{\begin{array}{ccc} 1-a_{s} & \text{if} & \left(r_{i}\leq 0.5\right)\wedge \left(a_{s}>0.5\right)\\ 1-r_{i} & \text{if} & \left(r_{i}>0.5\right)\wedge \left(a_{s}\leq 0.5\right)\\ 1-\frac{r_{i}+a_{s}}{2} & \text{else} \end{array} \end{array}$$ (6)

An agent's *Response efficacy* *e*_*R*_ refers to the perceived effectiveness of not smoking in preventing harm. If agents believe that abstaining from smoking will protect them from health risks, they are more likely to engage in adaptive behavior. The perception of harm is based on factors like perceived severity *s*_*p*_, perceived vulnerability *v*_*p*_, and the agent's attitude *a*_*o*_ toward smoking. If the consequences of smoking (such as health risks) are perceived to be severe, the agent is more likely to believe that quitting smoking is an effective measure to avoid harm. Similarly, if an agent feels vulnerable to these risks (e.g., due to poor health or high safety needs), they are more inclined to view quitting as a worthwhile protective action.


$$\begin{array}{l} e_{R}=a_{o}*\frac{\left(s_{p}+v_{p}\right)}{2} \end{array}$$ (7)

*Response costs* *c*_*R*_ are the perceived obstacles or challenges associated with adopting the adaptive behavior of quitting smoking. While high self-efficacy *e*_*S*_ and response efficacy *e*_*R*_ encourage quitting, response costs can discourage agents if they are perceived as too high. Equation 8 shows the two cases used to determine perceived response costs: if the need for belonging (*n*_*b*_) is rather low, the response cost is a combination of the opportunity costs (missing out on intrinsic reward *r*_*i*_) and the need for belonging which is not being satisfied. In case of a higher need for belonging, the cost of quitting is a combination of the missed-out-on intrinsic rewards and the perceived attitude of the environment (*a*_*s*_), which is perceived stronger by agents who have a strong desire to conform to their group.

As such, if an agent derives significant pleasure from smoking, the cost of quitting is perceived as higher, making it harder for the agent to quit. Likewise, the influence of the agent's social environment (friends and local community) also plays a role in shaping response costs. If the agent has a strong need for belonging, they may perceive quitting as a social risk, raising the response costs.


$$\begin{array}{l} c_{R}=\{\begin{array}{ccc} \left(r_{i}+n_{b}\right)*0.5 & \text{if} & n_{b}\leq 0.5\\ \left(r_{i}+a_{s}\right)*0.5 & \text{else} \end{array} \end{array}$$ (8)

As a result, the coping appraisal *A*_*c*_ is computed as follows:


$$\begin{array}{l} A_{c}=\frac{e_{R}+e_{S}}{2}-c_{R} \end{array}$$ (9)

#### 3.4.2 Resolution of cognitive dissonance

Intentions are strong behavioral predictors, but individuals often fail to act on them, leading to the intention-behavior gap. Acting contrary to intentions creates cognitive dissonance, which might lead to changes in behavior or attitude of individuals. Therefore, it is important to take the formation and pursuit of intention of agents into account during modeling.

To investigate this phenomenon, we investigate a modified PMT-model with amplified needs and compare it against the model presented by Kurchyna et al. (2024), which utilizes an unmodified PMT interpretation. When behavior conflicts with intention, cognitive dissonance *D* increases based on the disparity between coping and threat appraisal. Likewise, matching intentions and actions lead to a gradual decrease of cognitive dissonance, as summarized in Equation 10.


$$\begin{array}{l} D_{new}=\left\{\begin{array}{cc} D_{old}-|A_{c}-A_{t}| & \text{if Action \& Intention match}\\ D_{old}+|A_{c}-A_{t}| & \text{if not Action \& Intention match} \end{array}\right\} \end{array}$$ (10)

**Figure 4:**
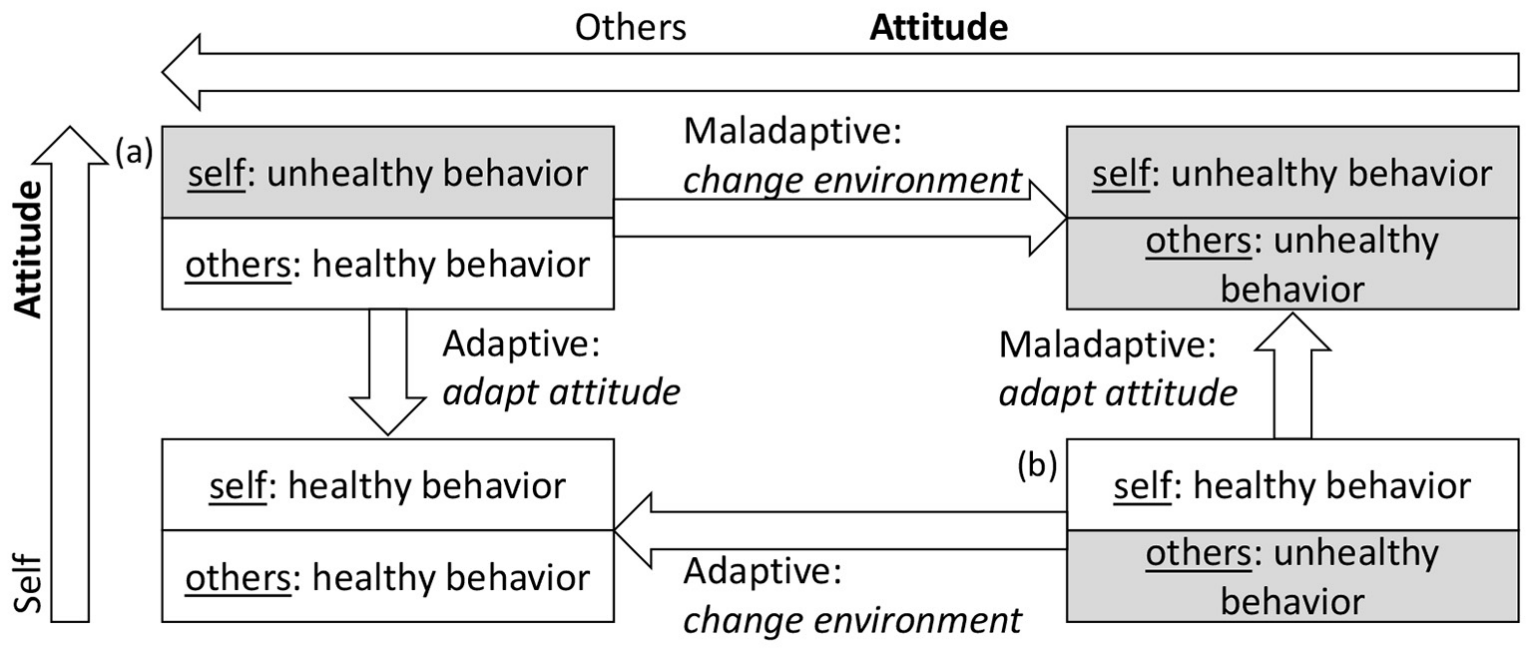
Resolution strategies for cognitive dissonance according to Kurchyna et al. (2024): adaptive actions lead to non-smoking agents seeking a non-smoking environment through cessation or environment change, while maladaptive agents lead to smokers grouping with other smokers.

Cognitive dissonance reduction strategies only activate when dissonance exceeds a certain threshold past which an agent will consider the current status untenable. Strategies include changing cognitions or creating new conflict-free conditions, as shown in Figure 4.

As first major strategy, *Change of Cognition* is an agents' attempt at conflict resolution by gradually adopting less dissonant attitudes. Instead of relying solely on social pressure to determine behavior as in the original model, the adjusted model emphasizes needs as the driver of behavioral change. When agents act contrary to intentions (e.g., choosing to smoke while not favoring smoking due to social pressure from smoking friends and/or strong needs for belonging), their attitude adapts to align with behavior, following the induced-compliance paradigm. This minimizes the gap between internal states and actions.

Alternatively, agents try to *Create New Consonsant Cognitions* by changing their environment. Since social pressure plays a key role in dissonance, agents attempt to reduce it by cutting ties with friends who exert excessive pressure. The contact termination threshold determines when agents replace friends based on attitude differences. Through the termination and formation of new friendships, agents change their environment to reduce social pressure.

Equation 11 summarizes the selection of actions, also depending on the two model parameters change rate *p*_*c*_ and contact termination threshold *p*_*t*_, further explained in Table 1.


$$\begin{array}{l} \text{Action}=\left\{\begin{array}{cc} \text{Nothing} & ,\text{if}\\ \left(\left|a_{o}-a_{s}\leq p_{c}\right|\right)\vee \left(a_{o}\geq 0.98\right)\vee \left(a_{o}\leq 0.02\right)\\ \text{Change Environment} & ,\text{if}\\ |a_{o}-a_{s}|\geq p_{t}\\ \text{Adapt Attitude} & ,\\ \text{otherwise.} \end{array}\right\} \end{array}$$ (11)

**Table 1:** Calibration-based parameters: Value ranges and recommended defaults according to Kurchyna et al. (2024).

**Parameter**	**Description**	**Range**	**Value**
*n_people*	Number of agents	int	100
*Conformists*	% of conformists	0 – 1.0	0.23
*Hedonists*	% of hedonists	0 – 1.0	0.64
*Self-Protectors*	% of health-aware	0 – 1.0	0.18
*pressure_mod*	Strength of perceived pressure	0 – 1.5	1.25
*contact_term* *p*_*t*_	Allowed difference between attitude and perceived pressure before changing environment	0 - 1.0	0.4
*min_duration*	Minimum number of days that must elapse before a need is reduced	0 – 20	3
*moving_distance*	move forward set number of units	1 – 5	2
*number_of_friends*	Number of peers each agent has	0 – 10	2
*change_rate* *p*_*c*_	Rate of change of attitude	0 – 0.1	0.01
*needs_multiplier*	Strength of the selected salient need	1 – 2	1.8
*gain_multiplier*	Action multiplier	0 – 1.0	0.9
*allowed_cog_diss*	Minimum level of cognitive dissonance must be achieved before attempting to reduce it	0 - 0.5	0.05

## 4 Simulation results and evaluation

During the simulation, where each step represents an abstract situation (without further specification as to time and setting), agents experience the urge to satisfy their needs. Individuals either feel compelled to smoke or are discouraged from smoking based on their salient needs. The extended model was re-calibrated and sensitivity analysis was performed before proceeding with experiments to explore the changes in smoking choices as well as observations of the intention-behavior-gap.

### 4.1 Experiment design and calibration

The experiment's primary aim is to examine how needs, social pressure, and PMT interact within a simulated agent-based environment. Using NetLogo (Willensky, 1999), the model includes agents with distinct behavioral archetypes, each influenced by parameters detailed in Table 1. Before running the simulation, these parameters, such as social pressure intensity, cognitive dissonance thresholds, and agent archetypes, need to be configured via calibration for the model to behave plausibly (see Table 1).

To ensure realistic outcomes, calibration was done through NetLogo's BehaviorSearch, which iteratively tests parameter combinations to align the model's behavior with empirical evidence. For instance, calibration aimed to match the simulated proportion of smokers to a target of 29%—based on German demographic data (Starker et al., 2022). Additionally, the target also included a reduction of inaction to capture dynamic interactions. Key calibration considerations included agent distribution among hedonists, conformists, and self-protectors, along with tolerance levels toward differing opinions (set by the contact term) and cognitive dissonance thresholds, which were set low to prompt dynamic adjustments. Given the large parameter space, these additional requirements were introduced to limit the pool of suggested configurations to potentially plausible sets of parameters. Without these additional requirements, alternative solutions to an optimization problem with only the number of smokers as target can yield an appropriate calibration with parameter values that are nonsensical given the concepts they represent.

It is important to note that the parameters utilized and calibrated in the original model by Kurchyna et al. (2024) were retained without adjustments to ensure a more accurate comparison between the two models. Consequently, only the parameters introduced during the extension's development were subjected to calibration. The target function was the same as used by Kurchyna et al. (2024):


$$\begin{array}{l} \mathrm{fi}tness=(\frac{\left|Smokers\right|}{\left|N\right|}-0.3*1000)+\\ \;\left(\sum do\text{\_}nothing*0.1\right)+\\ \;((|Conformists|+|Hedonists|+\\ \;|Security|)-1)*10 \end{array}$$ (12)

The fitness function targets to minimize the deviation of the smoker ratio from the target, which was rounded to 30%. The large multiplier prioritizes the correct amount of smokers over the other conditions and heavily punished deviation. Additionally, the number of inaction-decisions should be kept low—before the addition of this term, the calibration favored configurations in which agents start out in the correct ratio and remain static.

The pressure modifier amplifies the effect of pressure with a value of 1.25. Observations indicate that individuals tend to modify their environment only in cases of significant clash of attitudes, as indicated by the calibrated *contact_term* value of 0.4. This signifies a substantial level of tolerance toward differing opinions before active attempts to alter surroundings occur (Kurchyna et al., 2024). The parameter *min_duration* is a new addition in the extended model, specifying the minimum number of days required for a need to diminish. In contrast to the method used by Heidari et al. (2020), where needs diminish every tick, this implementation chooses a less frequent reduction cycle occurring every 3 ticks. This approach aims to prevent rapid depletion of needs. Due to limited spatial mobility and a small social network comprising only two friends, agents tend to exhibit a preference for maintaining their current environment over seeking new contexts. This preference is reinforced by a low change rate, encouraging gradual modifications rather than sudden, drastic shifts (Kurchyna et al., 2024). The parameter *needs_multiplier*, determining the strength of the selected need during need satisfaction and associated activity selection, is set to 1.8, emphasizing the greater importance of the chosen salient need. The success of actions carries significant weight, indicated by a value of 0.9. Additionally, a relatively low threshold for cognitive dissonance suggests that individuals quickly reach a state of discomfort, requiring more frequent efforts to reduce it.

During testing, the system typically stabilized after about 30 simulation steps, during which agents formed groups with homogenous behaviors, akin to patterns in established segregation models (Domic et al., 2011). After finalizing parameter values, the model's output was analyzed through sensitivity testing to better understand interactions between factors.

### 4.2 Sensitivity analysis

The extended model underwent a sensitivity analysis to assess parameter impacts, using PyNetLogo (Jaxa-Rozen and Kwakkel, 2018) and SALib (Herman and Usher, 2017) for Sobol indices computation, which measure the variance in model output attributable to each parameter. This analysis was compared with the initial model's outcomes. Findings showed a notable increase in alignment between intentions and actions, especially for smoking behavior, with initial character traits—Conformists, Hedonists, and Self-Protectors—strongly influencing individuals, choices. This observation aligns with real-world behaviors where people often remain consistent with their established attitude—for example, smokers continue smoking, health-conscious individuals avoid smoking (Høie et al., 2010).

**Figure 5:**
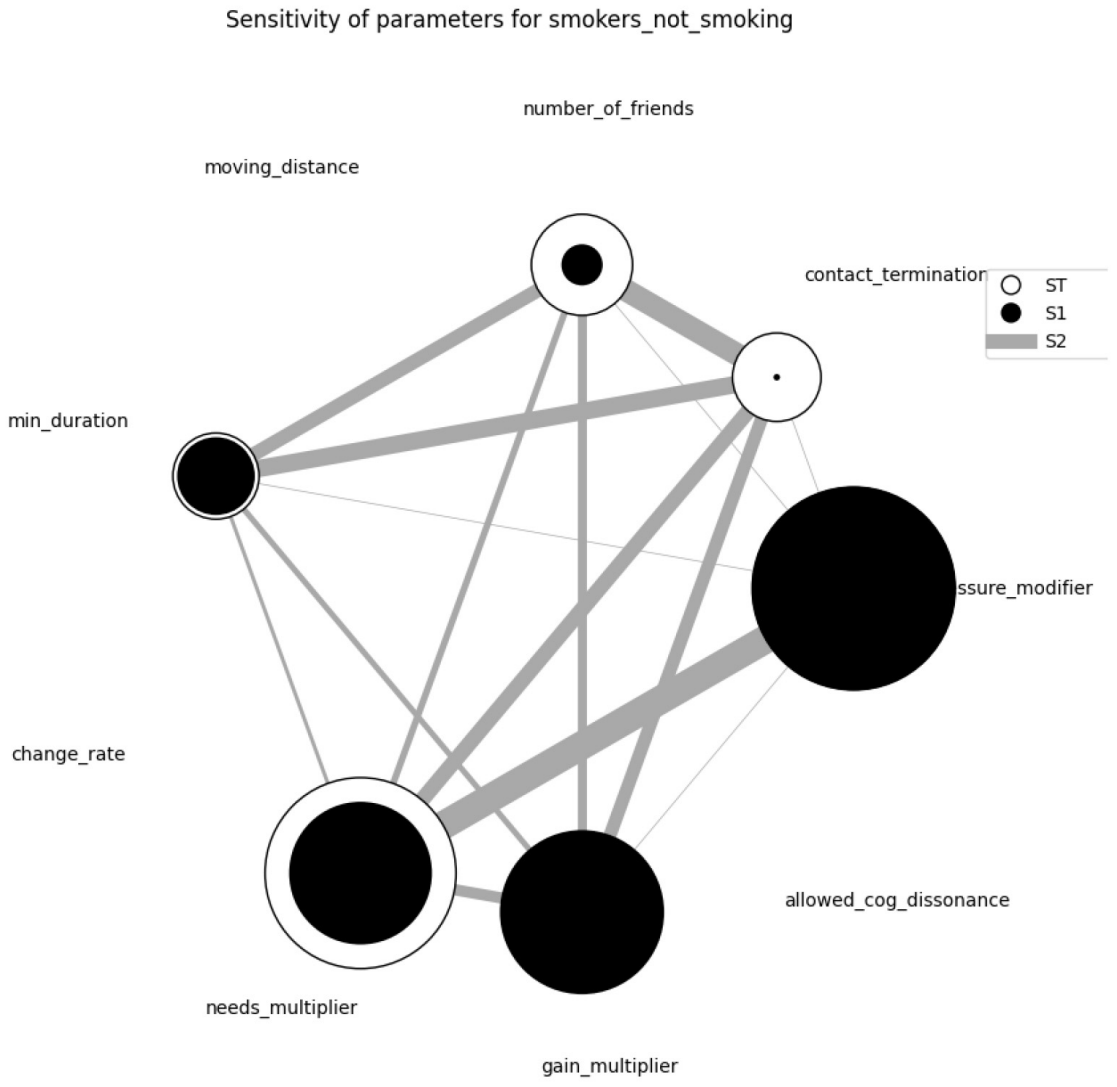
Sensitivity analysis on factors influencing smokers' abstention.

Figure 5 visualized the results of one of the sensitivity analyses which were performed, in this case an analysis of which inputs influence the abstention of smokers. The black circles (S1 indices) represent the individual contribution of a variable to the variance of the outcome - large black circles signify a strong impact of a variable. In addition to that, the outer circle (ST indices) denote contributions *in interaction* with other parameters, with the width of the connecting bars between variables (S2 indices) visualizing the connection strength between two parameters and their shared influence on the outcomes.

In the extended model, parameters tied to individual needs—the needs multiplier (reflecting the strength of a need) and the gain multiplier (affecting how actions satisfy needs)—proved crucial, as seen in the relative size of the respective circles. Self-actualization emerged as a dominant need, pushing individuals toward more polarized behaviors: strongly inclined either to smoke or to abstain. This differs from the original model, where social pressure played an even more significant role in guiding behavior. In the extended model, social pressure's influence weakened, giving way to needs-based motivations that encouraged more distinct, individualized behaviors. The pressure modifier remains a dominant parameter with strong interactions with the needs, but is no longer as absolute as presented in the original paper (Kurchyna et al., 2024).

### 4.3 Observing the intention-behavior-gap: a comparison

The central focus of this experiment is not to create a realistic simulation of smoking and quitting. Instead, its main purpose is to serve as a case study to examine the interaction between needs, social pressure, and the PMT. As a result, the analysis and experiments are conducted with this specific objective in mind.

The analysis focused on three distinct categories of output variables:

- *Intention-behavior alignment:* The frequency with which individuals, favoring smoking as indicated by their intention to smoke in contrast to the smoking attitude in the original model (Kurchyna et al., 2024), ultimately act upon their intentions regarding smoking is analyzed by applying the PMT.
- *Smoking behavior:* The number of habitual smokers (smoking attitude≥0.75), occasional smokers (0.25 < smoking attitude < 0.75), and convinced non-smokers (smoking attitude ≤ 0.25) in the population, as well as the number of actual smokers and non-smokers in the extended model, represented by observed behaviors from the PMT.
- *Actions:* The frequency with which agents choose actions such as *do nothing, change environment*, and *adapt attitude*.

All experiments were performed with 100 repetitions to account for stochastic influences.

In the following figures, the results of a computation *without* needs are displayed in red — this is analogous to the results that would have been obtained by the original model by Kurchyna et al. (2024). The needs-adjusted results are portrayed in green.

**Figure 6:**
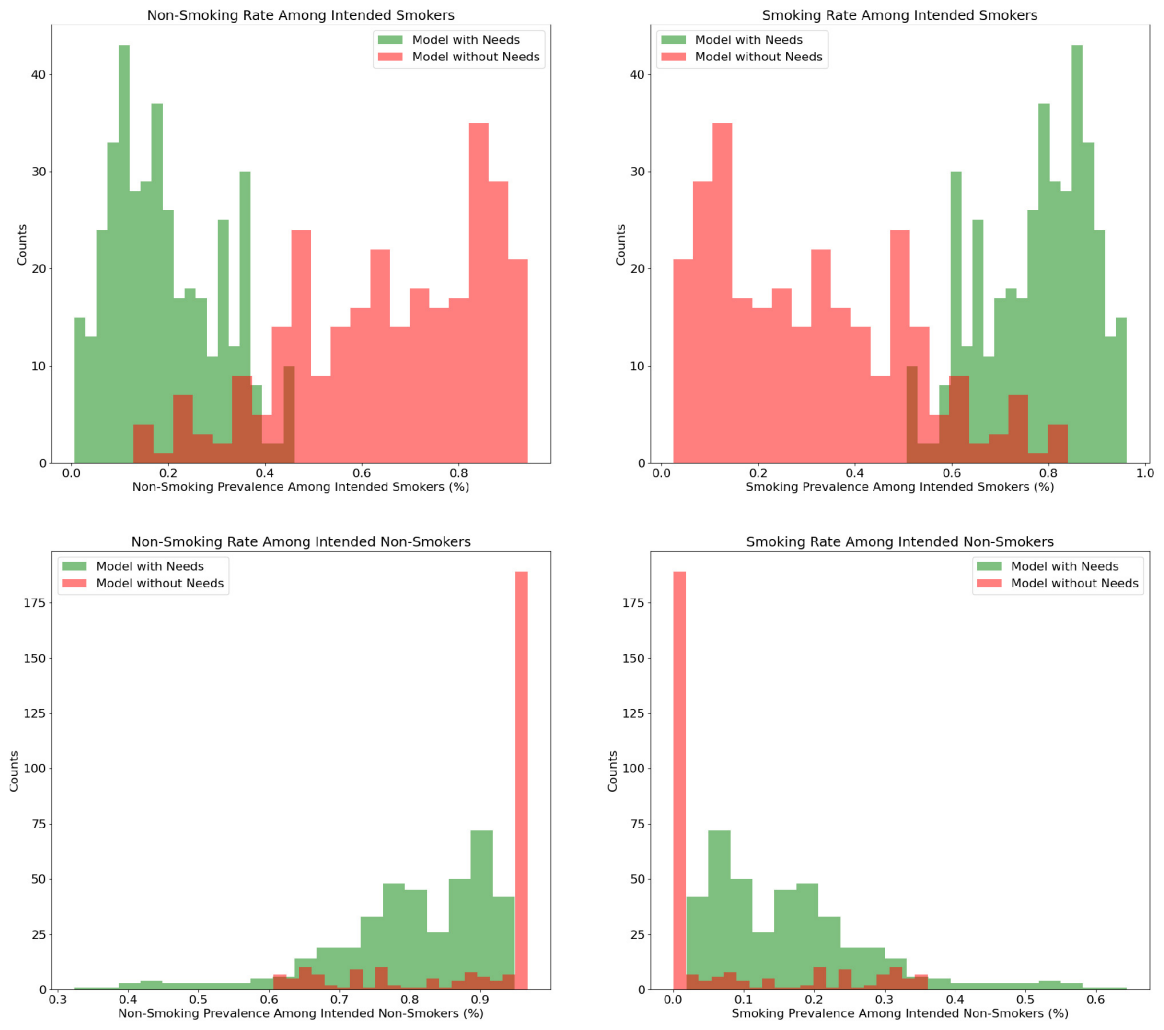
Comparison of modeling approaches: aligning intentions with behaviors.

Figure 6 shows a noticeable increase in individuals who act according to their intentions in the extended model. For example, smokers intending to smoke were more likely to follow through, while those with a strong intention not to smoke typically resisted smoking. Non-smokers with extreme attitudes appeared to be more sensitive to the needs multiplier, indicating that polarized attitudes toward smoking were influenced more by individual needs than social factors.

Notably, the model explicitly examining individual needs displayed a substantially lower ratio of individuals experiencing the desire to smoke but resisting the urge compared to the model not directly exploring needs. In the initial model, there is a significant difference in the percentage of individuals translating their smoking intentions into actions compared to the extended model. However, among those intending not to smoke, a significant majority consistently adhered to their decision in both models. While the extended model indicates slightly lower adherence among individuals without a desire to smoke, the overall trend remains similar. This observation is further reflected in the rate of smoking among those originally intending not to smoke, where the initial model depicts a slightly lower rate compared to the extended model, although this difference is not notably meaningful. While the initial approach emphasizes social pressure, represented by the pressure modifier, as the primary influence on the observed variables (Kurchyna et al., 2024), the extended model underscores the increased significance of needs, diminishing the influence of social pressure. The amplified impact of social pressure in the original model could clarify why more individuals decide against smoking, as the initial population strongly leans toward abstaining from smoking.

**Figure 7:**
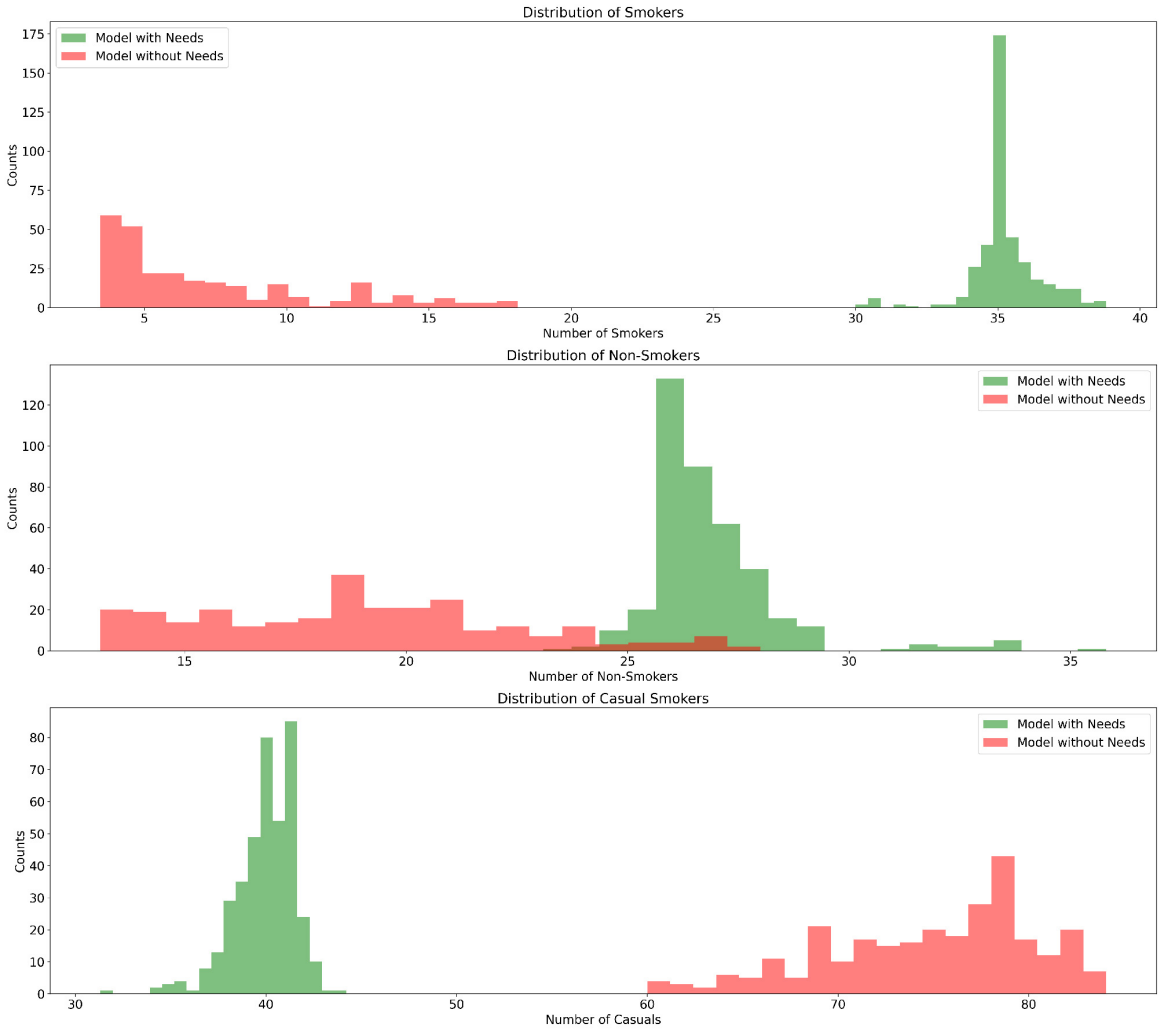
Model comparison: smoking distribution analysis.

Another observed outcome is the increased proportion of both smokers and non-smokers in the extended model, which diverges from general global trends indicating a decline in smoking rates (see Figure 7). Here, extreme attitudes and needs drive behavior. Strong interactions among parameters like needs, the gain multiplier, and social environment variables led to a broader range of behaviors, reducing the role of cognitive dissonance reduction as a sole motivation.

This is similar to the initial expectation that the introduction of needs related to safety or pleasure might lead individuals to act according to their dominant needs and either have a relatively low attitude toward smoking or a high one. The shift toward more extreme behavior is consistent with the findings of Resta et al. (2023), demonstrating that when one need predominates, creating an imbalance and potentially neglecting other needs, it leads to extreme behaviors such as smoking and non-smoking.

During further experiments, the impact of the needs-multiplier as central aspect was investigated - the reduction of this parameter led to a decrease of smokers and those whose actions mismatch their intentions. Without the reinforced needs (*needs*_*multiplier* = 1.2), social pressure is the primary driver of decisions, leading to a stronger homogenization of attitudes. When the needs are strongly affecting agents' judgement (*needs*_*multiplier* = 2.4), the polarization of groups is more pronounced. For values below 1.3, the dedicated smokers and convinced non-smokers intersect within 150 model steps and show strong trends toward full homogenization into a non-smoking population. This confirms the assumption, that needs drive the observed polarization.

## 5 Discussion of results

According to Ghorbani et al. (2021), it is human needs that drive behavior. Needs play a pivotal role in determining compliance with formulated intentions, significantly impacting individuals' decisions concerning smoking. Consequently, the integration of human needs into this model provides a nuanced understanding of decision-making processes and their correlation with behaviors. Within this model focusing on human needs, agents continually pursue a balanced fulfillment. This pursuit of equilibrium can be influenced by the social environment, which can either facilitate or hinder their quest for stability. The structured extension of the PMT, considering various human needs and utilizing a two-layered approach to contact networks, proves adaptable to diverse scenarios involving agents navigating uncertain situations and conflicting goals. Addictions, both physical and psychological, are use cases where a needs-based approach may be more appropriate to explain how addiction skews the subjective perception of risks and benefits and, finally, override rational choice.

In the larger context of ABMs as means of explaining observations in the real world (Len-Medina, 2017), the inclusion of needs improves the explanation for smoking dynamics in terms of the Both models were calibrated to the same target to establish face-validity, and the original model by Kurchyna et al. (2024) suggests that undecided people will be pulled into either direction by their surroundings, while most smokers and non-smokers remain by their position persistently–which is not wrong, the model is, however, insufficient to explain the high failure rate of smoking cessation and the power of peer pressure.

By including needs which can amplify dissonant conditions, we can observe the formation of the intention-behavior gap more clearly, with long-term intentions in the psychological sense typically formulated through rational reasoning, and behavior arising spontaneously and influenced by immediate contributors. Compared to other alternatives, such as pure utility-maximization, the needs-based approach preserves the seemingly irrational nature of human decision making—choices *make sense* in the moment, even if they are counter to well-reasoned intentions for long-term goal achievement.

Both failed smoking cessation (as need for pleasure or social belonging) and peer pressure (need for social belonging) can be explained through the combination of needs and values as sources of heterogeneity in how agents evaluate the external factors they are exposed to.

However, the relationship of individual actions and need levels is highly hypothetical, and subject to human error introduced through calibration and other decisions during operationalization unrelated to the subject matter at hand, such as the size of the personal social network and range of local contacts. The risk of divergent results, similar to Muelder and Filatova (2018), is particularly high when using theoretical frameworks without empirical data to back it up. As such, any serious use case requires not only empirical data as support, sparse as it may be, but also relevant expertise to validate assumptions and decisions not only from a purely mechanistic perspective, but also embedded in the overall purpose of the simulation study and the questions that are meant to be answered.

## 6 Conclusion and outlook

The integration of needs into behavior modeling holds significant implications for comprehending and predicting human behavior. Embedding needs within the components of the PMT allows for a more nuanced representation of individual behaviors in health-related contexts. Needs facilitate modeling individual personalities with diverse preferences. Human needs exert a pivotal influence on determining adherence to or deviation from intended actions. Their integration promotes a heightened alignment between intention and behavior. Furthermore, needs contribute to the manifestation of more extreme behaviors, as evidenced in the investigated case, resulting in an increased count of both smokers and non-smokers.

To enhance the ecological validity of the simulation, a significant avenue for future work involves the integration of physiological feedback loops. Although omitted in the current model to maintain a clear focus on agent interaction, the inclusion of nicotine-driven physiological needs is essential for capturing the compulsive nature of smoking (Benowitz, 2010), as it will compete with other needs and thus counteract the intention to stop smoking.

Potential future research could aim to validate the applicability of the model in diverse health-related domains. Drawing on existing literature, for example, Di Tosto and Dignum (2012) assumed that competing needs cannot be met simultaneously. Subsequent research could advance this by not only initializing these needs and their associated values randomly, but rather basing them on conflicting values. Consequently, an individual with a high value for security would inherently possess a high threshold for safety, accompanied by comparatively lower values for hedonistic factors. This nuanced approach could enhance the understanding of how conflicting needs shape behavior within various health-related contexts.

More immediate upcoming tasks involve validating the model with empirical data and incorporating it into the initialization of agents. Currently, agents' values remain constant throughout the simulation due to complexity constraints (Van de Poel, 2021) and time span (Bardi et al., 2009). In future research, there is potential to introduce dynamic components for these values, allowing them to change over the course of the simulation. Correspondingly, the threshold values could dynamically adjust to adapt to changing situations with variable thresholds. This adjustment would introduce additional dynamic in decision-making behavior, resulting in even more realistic behavior. Additionally, the overall mobility of the agent significantly contributes to portraying a dynamic and realistic system behavior. This can be further improved by incorporating realistic movement routines and forming habits that include random elements, facilitating unexpected encounters (Kurchyna et al., 2022).

Furthermore, it is noteworthy that the incorporation of psychologists' expertise enhances the authenticity of the behavioral model, resulting in a more nuanced and precise depiction. Finally, future studies could explore a wider array of human needs, as this study only examined three needs for the modeling process. Additionally, this concept can be applied to other use cases by modifying the available actions and their impact on different needs.

The primary contribution of this work involves integrating a selected group of needs into the components of the PMT. By incorporating these needs, the study offers a deeper understanding of how fulfilling particular needs can impact decision-making processes, leading to adaptive or maladaptive behaviors. The developed model exhibits considerable potential and, considering the listed possibilities for further development, can serve as a foundational framework for future decision-making in crisis.

## Data Availability

The raw data supporting the conclusions of this article will be made available by the authors, without undue reservation.
